# Response of characteristic hormones in tea roots and leaves under magnesium regulation and their balancing regulation on growth and quality

**DOI:** 10.3389/fpls.2025.1703380

**Published:** 2026-01-05

**Authors:** Xiaoli Jia, Yulin Wang, Tingting Wang, Bitong Zhu, Qiqi Weng, Yankun Liao, Junbin Gu, Yangxin Luo, Qi Zhang, Jianghua Ye, Haibin Wang

**Affiliations:** 1 College of Tea and Food Science/Fujian Key Laboratory of Big Data Application and Intellectualization for Tea Industry (Wuyi University), Wuyi University, Wuyishan, China; 2 College of Life Science, Longyan University, Longyan, China

**Keywords:** characteristic hormone, growth index, Mg ion, quality index, tea plant

## Abstract

Magnesium (Mg) plays a critical role in tea plant growth, profoundly affecting their development and leaf quality. In our study, we used hormone metabolomics technology to identify characteristic hormones that underwent significant changes in roots and leaves of tea plant when subjected to variations in Mg contributions. The results showed that characteristic hormones in leaves regulated by Mg were L-phenylalanine and indole, which positively regulated leaf resistance (0.942**), nutrient accumulation capacity (0.935**), photosynthesis capacity (0.917**) and plant height (0.921**). The characteristic hormones of the root were L-tryptophan, L-phenylalanine, indole and 2-methoxycarbonylphenyl beta-D-glucopyranoside, which positively regulated root resistance (0.940**), root vigor (0.948**), nutrient accumulation capacity (0.963**) and root growth index (0.917**). In contrast, exogenous treatments of characteristic hormones obtained from roots and leaves all effectively promoted tea quality and tea plant growth. It is obvious that with the increase of Mg concentration, tea plants synthesized a large number of characteristic hormones, which promoted their rapid growth, but reduced leaf quality, while exogenous treatment of the characteristic hormones to tea plants could promote their growth, and also improve leaf quality. The research provides valuable insights for achieving high-yield and high-quality cultivation for tea plants.

## 1 Introduction

China, globally recognized as the birthplace of tea, has a tea industry that holds a pivotal role in propelling agricultural development and rural revitalization. As of 2023, the expanse of tea plantations in China had reached 3,433,200 hectares. The aggregate output of dry raw tea hit 3,339,500 tons, yielding an output value of 329,668 million yuan. The core objective of tea cultivation is to harvest the delicate young buds and leaves of tea plants. Consequently, a substantial quantity of fertilizers are applied during the planting and management processes to ensure a stable and uninterrupted tea yield (Ye et al., 2023). Nitrogen, phosphorus and potassium are crucial in tea plant growth. Numerous scholars have carried out in-depth research in this area (Wei et al., 2022; Xie et al., 2022; Maity et al., 2022). Mg is one of the 16 essential elements that are crucial for plant growth (Javed et al., 2022). Although plants require less Mg than nitrogen, phosphorus and potassium, Mg plays a crucial role in regulating plant growth (Kleczkowski and Igamberdiev, 2021). Mg triggers the synthesis of plant genetic material and regulates the respiratory metabolic capacity of plants, thereby exerting an impact on plant metabolism (Ge et al., 2022; Kleczkowski and Igamberdiev, 2023). Mg directly influences plant chlorophyll synthesis and regulates the photosynthetic capacity of plants (Kan et al., 2022). Previous studies have shown that Mg promotes crop growth and nutrient utilization, such as increasing navel orange yield and sucrose content (Liu et al., 2022b), but also enhances soybean absorption efficiency of multiple elements (Lu et al., 2022). In tea plants, Mg directly participates in quality formation by regulating key gene expression related to secondary metabolism, amino acid metabolism, and chlorophyll synthesis, thereby affecting leaf development and quality foundation. Furthermore, Zhang et al. (2023b) found that Mg can significantly activate sugar metabolism processes through metabolomics analysis, promote the accumulation of sugars in tea, and physiologically confirm its function in quality construction. These findings collectively indicate that Mg plays a multidimensional and deep-seated regulatory role in the growth process of tea plants. Therefore, the system reveals the intrinsic mechanism of Mg affecting tea plant growth, which has important scientific significance for optimizing tea plant cultivation and precision fertilization strategies.

In the process of plant cultivation, numerous endogenous plant hormones exist, each playing a crucial role in regulating an extensive array of physiological and metabolic processes, thereby exerting a significant influence on plant growth (Waadt et al., 2022). Take salicylic acid and jasmonic acid for example, these hormones are involved in modulating plant resistance to environmental stresses, enhancing plant resistance and increasing plant yield (Liu et al., 2022a). Conversely, growth hormone is intricately associated with regulating the germination of apical buds and increases the intensity of photosynthesis and respiratory metabolism within plants (Batista-Silva et al., 2024). Cytokinins are advantageous in expediting the development of lateral branches and enhancing plant productivity (Sosnowski et al., 2023). The synthesis of endogenous plant hormones is intricately linked to the plant’s capacity to absorb and accumulate Mg. Mg has an impact on the intensity of gene expression within metabolic pathways of plant hormone, thus alters the ability to synthesize endogenous plant hormones, ultimately having an effect on plant growth (Du et al., 2024). For example, Mao et al. (2022) revealed that Mg significantly improved the content of indole-3-acetic acid and zeatin in Chinese broccoli, thus led to an enhancement of the photosynthetic and antioxidant capacity, promoted its growth, and increased its yield. He et al. (2024) explored the impact of exogenously applied Mg solutions on maize growth and found that Mg stimulated the production of gibberellin and indole-3-acetic acid in maize, fortified maize resistance, and augmented its yield. It is obvious that there exists a close association between Mg’s influence on plant growth and the capacity for synthesizing endogenous plant hormones.

It is postulated that Mg’s impact on tea plant growth is associated with its hormone synthesis capacity, and that Mg regulation triggers alterations in the synthesis of characteristic endogenous hormones of the tea plant, thereby modulating both its quality and growth. Accordingly, this study used different concentrations of Mg ions to treat hydroponically grown tea plants, to determine the growth indexes, photosynthetic physiological indexes, growth indexes, antioxidant capacity and tea quality indexes, and subsequently to analyze Mg’s impact on tea plant growth and leaf quality. At the same time, tea plant roots and leaves were used to measure hormone metabolomics, and characteristic hormones that underwent significant changes were obtained under the regulation of Mg. Finally, the exogenous treatment of tea plants with characteristic hormones was used to verify the effect of characteristic hormones on leaf quality and tea plant growth. This study explores the change of characteristic hormones and their effects on leaf quality and tea plant growth after Mg regulation, which is meaning for the exogenous regulation of hormone to leaf yield and quality, tea plant growth.

## 2 Materials and methods

### 2.1 Experimental design and sample sampling

In this research, the Rougui variety of *Camellia sinensis* tea plants was selected for hydroponic cultivation. The focus was on experimental investigations regarding magnesium (Mg) regulation, for which the Mg concentration in the culture solution was precisely adjusted. Uniform one-year-old tea plant seedlings were carefully chosen. These seedlings had an approximate height of 35 cm and a diameter of 0.3 cm. Prior to transplanting the seedlings into a complete nutrient solution, their roots underwent meticulous and thorough washing with deionized water. The nutrient solution was formulated according to the method described by Sun et al (Sun et al., 2020), and its pH value was set at 4.5. The primary components of the nutrient solution included KNO_3_ (125 μmol/L), (NH_4_)_2_SO_4_ (187.5 μmol/L), KH_2_PO_4_ (100 μmol/L), K_2_SO_4_ (25 μmol/L), CaCl_2_ (100 μmol/L), MgSO_4_ (100 μmol/L), FeSO_4_ (16 μmol/L), Al_2_(SO_4_)_3_ (200 μmol/L). Subsequently, the seedlings were immersed in this solution for 45 days. This incubation period was designed to ensure that the seedlings could fully recover from the transplantation and resume normal growth.

Once the tea plant seedlings had restored their growth, they were carefully removed. Subsequently, their roots were gently rinsed with deionized water. Following this, the seedlings were transplanted into nutrient solutions featuring different Mg concentrations for treatment, with 12 plants in each pot. To ensure the accuracy and reliability of the experimental results, each treatment was independently replicated three times. Based on the previous research (Zhang et al., 2023), in this study, three distinct Mg ion concentrations were set: 0 mmol/L, 0.4 mmol/L, and 0.8 mmol/L. These concentrations were respectively designated as M0, M1 and M2, respectively. The nutrient solution was prepared in the manner previously described, with the sole alteration being the adjustment of the Mg ion concentration. Following transplantation, the tea plant seedlings were nurtured within a greenhouse. The greenhouse conditions were precisely regulated as follows: the light intensity was set at 1500 lux, the photoperiod spanned 12 hours (8:00 ~ 20: 00), the temperature was consistently maintained at 25°C and the humidity was held at 75%. Over a period of 21 days, the tea plant seedlings underwent treatment with culture solutions featuring varying Mg ion concentrations. Throughout this duration, the culture solution was continuously aerated around the clock, ensuring optimal oxygen supply. Additionally, the nutrient solution of each corresponding concentration was refreshed once every seven days. Upon the completion of the treatment phase, multiple key parameters of the tea plant seedlings were meticulously measured. These included plant height, photosynthetic physiological indexes, dry weight, and root morphological indexes. Simultaneously, samples of tea plant roots and leaves were carefully collected and promptly immersed in liquid nitrogen. These samples were earmarked for subsequent in-depth analysis of physiological indexes associated with the tea plant’s resistance mechanisms, quality-related indexes, and hormone metabolome.

### 2.2 Determination of tea plant growth indexes

Tea plant growth was evaluated through measurements of plant height, root morphology and dry weight. To ensure accuracy, each treatment was replicated three times for accuracy. Plant height was measured using a tape measure. The measurement was taken from the junction of the roots and the stem to the highest point of the plant. For the assessment of root morphology, a root scanner (Expression 1200XL, Epson, Suwa, Japan) was employed (Zhu et al., 2022). Specifically, the tea plant root system was placed in a transparent root tray and the optical resolution of the scanner was set to 2400×4800 dpi for scanning. Subsequently, the scanning results were analyzed using WinRHIZO Pro 2019a software (Regent Instrumengts Inc, Canada) to acquire the data on different tea plant root indexes of the tea plant seedlings. Dry weight was determined by the drying method. Firstly, the entire tea plant seedlings were placed in an oven at 120°C for 15 min. Then, they were dried at 80°C until a constant weight.

### 2.3 Determination of photosynthetic physiological indexes and fluorescence parameters in tea plant

The photosynthetic physiological indexes of tea plant leaves were evaluated using a chlorophyll analyzer (SPAD-502 PLUS, Tokyo, Japan) and an LI-6400XT Portable Photosynthesis System (Li-Cor, Lincoln, NE, USA). The inverted two leaves (functional leaves) of tea plants were selected to measure indexes related to photosynthesis with five replicates per treatment. The chlorophyll analyzer was utilized to measure quantify the chlorophyll content in the leaves. The LI-6400XT System was employed to measure multiple parameters of tea plant leaves, including intercellular CO_2_ concentration, net photosynthetic rate, stomatal conductance, net photosynthetic rate and photosynthetic water use efficiency. These measurements were conducted between 9:30 and 11:30 on a sunny day. During these measurement process, the leaf temperature was maintained within the 25°C to 26°C range, the ambient CO_2_ concentration was set at 360 ppm, the vapor pressure deficit in the vessel was kept below 1 kPa, and the photon flux density was set at 1500 μmol/m^2^·s. In addition, fluorescence parameters of tea plant leaves, including the actual photochemical efficiency of PSII in the light, maximal quantum yield of PSII, minimum fluorescence under light, maximum fluorescence under light, and quantum yield of PSII electron transport, were determined using a PAM-2500 chlorophyll fluorometer (WALZ, Germany). For these fluorescence parameter measurements, each treatment also had five replicates. Prior to measurement, tea plant leaves were pre-acclimated in a dark environment for 30 min. During the measurement, the saturated pulsed light was set to 8000 μmol/m^2^·s, and the measuring light intensity was kept at less than 0.05 μmol/m^2^·s.

### 2.4 Determination of antioxidant enzyme activities and nutrient content of tea plant roots and leaves

The antioxidant enzyme activities of tea roots and leaves were examined using an Enzyme Linked Immunosorbent Assay Kit provided by Shanghai Preferred Biotechnology Co., Ltd. The research primarily aimed to measure the activities of superoxide dismutase (SOD), peroxidase (POD) and catalase (CAT). For each sample, the assay was replicated three times to ensure accuracy and reliability. Specifically, 0.3 g of fresh tea plant leaves or roots were precisely weighed and subsequently extracted using appropriate kits. Next, the absorbance of these extracts was measured by a BioTek Synergy2 Gene 5 multifunctional enzyme-labeling instrument (Vermont, USA).

The activities of SOD, POD, and CAT were measured at wavelengths of 560, 470, 240 nm, respectively. These activities were expressed as units per gram (U/g) of the respective enzymes. Regarding the determination of the nutrient content in tea plant leaves and root system, it was conducted with reference to “Experimental instruction in plant physiology” (Wang, 2006). The main nutrients under measurement were nitrogen, phosphorus and potassium, and each sample was analyzed with three replicates. Briefly, the collected tea plant leaves or roots were dried separately, finely ground, and passed through a 60-mesh sieve. Then, 0.3 g of the sample was carefully placed in a digestion tube, and 5 mL of H_2_SO_4_ was added. The mixture was subjected to digestion at 170°C for 30 min, followed by further digestion at 300°C for 10 min. Subsequently, 1 mL of 30% H_2_O_2_ was added, and digestion was continued for another 10 min. After allowing the mixture to cool, the volume of the solution was adjusted to 100 mL. This solution was then utilized to determine the nitrogen, potassium, and phosphorus content. The nitrogen content was quantified via the Kjeldahl method. To determine the phosphorus content, the molybdenum antimony resistance colorimetric approach was employed. Regarding the measurement of potassium content, flame atomic absorption spectrophotometry was utilized.

### 2.5 Determination of tea quality indexes

Tea quality was mainly evaluated through a variety of indexes, such as g water extract, tea polyphenols, total catechins, theanine, free amino acids (excluding theanine), caffeine, flavonoids, and soluble sugars. The process began with tea leaves being heat - treated to deactivate enzymes. First, the leaves were subjected to a temperature of 105°C for fixation and then dried at 80°C until their weight remained constant. Afterward, the dried leaves were ground and passed through a 60-mesh sieve.

These processed leaf samples were then used to assess the above-mentioned quality indexes. Among these indexes, tea polyphenols and total catechins were measured using folinol colorimetry and gas chromatography, following the national standard of the People’s Republic of China (GB/T 8313-2018) (General Administration of Quality Supervision, Inspection and Quarantine of the People’s Republic of China, 2018). Theanine content was measured by high performance liquid chromatography, in accordance with the national standard of the People’s Republic of China (GB/T 23193-2017) (General Administration of Quality Supervision, Inspection and Quarantine of the People’s Republic of China, 2017). Caffeine was determined by UV spectrophotometry referring to the national standard of the People’s Republic of China (GB/T 8312-2013) (General Administration of Quality Supervision, Inspection and Quarantine of the People’s Republic of China, 2013a). Free amino acids were also determined by UV spectrophotometry, following the national standard of the People’s Republic of China (GB/T 8314 -2013) (General Administration of Quality Supervision, Inspection and Quarantine of the People’s Republic of China, 2013b). The free amino acid content was obtained by subtracting the theanine content from the total amount of free amino acids measured. In addition, water extract content was determined by hot water extraction. Soluble sugar content was measured using the anthrone colorimetric method, and flavonoid content was determined by aluminum trichloride colorimetric method. These three methods were carried out according to the procedures described by Wang et al. (2020).

### 2.6 Metabolomics analysis of hormones, hormone precursors and their derivatives in the roots and leaves of tea plants

To determine the hormones, hormone precursors and their derivatives (General term: Hormones) content in tea plant roots and leaves, 50 mg of fresh tea plant leaves or roots were first weighed. Then, these samples were combined with 1.5 mL of an extractant. The extractant was formulated with methanol, formic acid, and water in a ratio of 15:1:4. The resulting mixture was subjected to vortexing for 10 min. After that, it was centrifuged at 12,000 r/min at a temperature of 4°C for 5 min. Next, the supernatant was carefully collected and its volume was adjusted to 100 μL using 80% methanol. Finally, the solution was filtered through a 0.22 μm membrane and used for the determination of hormone content (Li et al., 2016; Floková et al., 2014).

The quantification of the hormone content was achieved by employing liquid chromatography tandem mass spectrometry. An Ultra Performance Liquid Chromatography system (ExionLC™ AD, manufactured by AB Sciex, Concord, Canada) was used in combination with a Tandem Mass Spectrometry instrument (QTRAP^®^ 6500+, AB Sciex, Concord, Canada). For the liquid chromatography part, a Waters ACQUITY UPLC HSS T3 C18 column (1.8 µm, 100 mm×2.1 mm i.d.) was utilized. Regarding the mobile phase, phase A consisted of ultrapure water containing 0.04% acetic acid, and phase B was acetonitrile with the same acetic acid concentration. The gradient elution protocol was programmed to initial with a 95% phase A composition at 0 min, gradually decreasing to 5% over the span of 0 to 8 min, and then linearly increasing back to 95% between 8 and 9.1 min. The chromatography column was kept at a temperature of 40 °C, with a flow rate set at 0.35 mL/min. Additionally, the sample injection volume was precisely measured at 2 μL. The operational parameters of the mass spectrometer encompassed an electrospray ionization temperature set at 550°C, with mass spectral voltages of -4500 V and 5500 V for negative and positive ion modes, respectively. Additionally, a collision gas pressure of 35 psi was employed. In the detection process, each ion pair was scanned and identified in accordance with their optimized declustering potential and collision energy (Šimura et al., 2018). To establish the standard curves for various hormone standards, 10 gradient concentrations spanning from 0.01 to 500 ng/mL were prepared for each hormone. These concentrations were then assayed using the aforementioned method, yielding chromatographic data corresponding to different hormone concentrations. These data were utilized to plot the standard curves, as detailed in **Supplementary Table S1**. Subsequently, the hormone content within the sample was ascertained by referring the standard curves.

### 2.7 Effects of exogenous treatments of characteristic hormones on the quality and growth of tea plant seedlings

Upon analyzing the mentioned above experimental results, it was found that 2 characteristic hormones, L-phenylalanine and indole, exhibited significant changes in tea leaves following Mg regulation. Meanwhile, 4 characteristic hormones, namely L-tryptophan, L-phenylalanine, indole and 2-methoxycarbonylphenyl beta-D-glucopyranoside, demonstrated significant changes in tea roots. Based on the content of these characteristic hormones, the corresponding concentrations of hormone solutions were formulated to treat tea plant seedlings. The seedlings that did not receive hormone treatment served as the control. The concentrations of the characteristic hormone treatments were detailed in **Table 1**.

**Table 1:** Treatment concentrations of characteristic hormones.

	Characteristic hormone	Concentration (mg/L)		
		Low	Medium	High
Leaf	L-phenylalanine	2.60	2.67	3.25
	Indole	9.10	10.70	10.90
Root	L-tryptophan	9.30	12.80	16.40
	L-phenylalanine	3.92	5.78	6.18
	Indole	0.71	1.01	1.23
	2-Methoxycarbonylphenyl beta-D-glucopyranoside	0.10	0.12	0.16

Tea seedlings with uniform growth and plant height were selected and transplanted to a complete nutrient solution for a period of 45 days to ensure their normal growth. This nutrient solution was periodically replaced with fresh complete nutrient solution. The exogenous application of characteristic hormones to tea plant leaves involved spraying the leaves with different concentrations of L-phenylalanine and indole. The control received spray treatments of distilled water, and each treatment was independently replicated three times. The hormone solution was evenly sprayed onto the top and bottom surfaces of the leaves until they were thoroughly wet, with each pot receiving approximately 80 mL of solution each time. This spraying process was repeated every 7 d for a total of 21 d, resulting in three applications in total. For the exogenous application of characteristic hormones to the root system, the concentration of L-tryptophan, L-phenylalanine, indole, and 2-methoxycarbonylphenyl beta-D-glucopyranoside in the hydroponic solution was adjusted according to **Table 1**. Each treatment was independently replicated three times. During the 21-day period, the culture solution was changed every 7 d, and characteristic hormone concentrations were readjusted as specified in **Table 1**.

After treating tea plant seedlings with characteristic hormones for 21 d, various growth indexes and leaf quality indexes were measured to assess the impact of these hormones. Specifically, the plant height, dry weight and net photosynthetic rate of the leaves were determined to understand how the hormones influenced the seedlings’ growth. Additionally, leaves were collected from the seedlings to measure the content of tea polyphenols, free amino acids and caffeine, thus analyzed the effects of characteristic hormones on tea quality. Specific methods used for these measurements have been described previously.

### 2.8 Statistical analysis

The raw data was initially processed using Excel 2020 for fundamental statistics and analysis. Statistical significances between different samples and between different indexes were assessed using paired Student’s t-tests, with a significant level set at *p* < 0.05. For further analysis and visualization, Rstudio software (v4.2.3) and various R packages were utilized (Wickham, 2019). Specifically, the gghalves (version 0.1.4) was used to create box plots for different samples with different indexes. The pheatmap package (version 1.0.12) was employed to generate heat maps. For principal component plots and volcano plots depicting hormone content differences between samples, the ggbiplot (version 0.55) and ggplot2 package (version 3.5.0) were used, respectively.

Orthogonal partial least squares discrimination analysis (OPLS-DA) models were constructed using the ropls and mixOmics. To analyze the weight of key hormones in distinguishing between different Mg treatment concentrations, the technique for order of preference by similarity to ideal solution (TOPSIS) was applied using the dplyr (version 1.1.4). Additionally, redundancy analysis, correlation interactions networks and partial least squares structural equation modelings (PLS-SEM) equations were constructed using vegan package (version 2.6.4), linkET package (version 0.0.7.1), and plspm(version 0.4.9), respectively.

## 3 Results

### 3.1 Effect of Mg regulation on growth and photosynthetic indexes of tea plant

**Figure 1:**
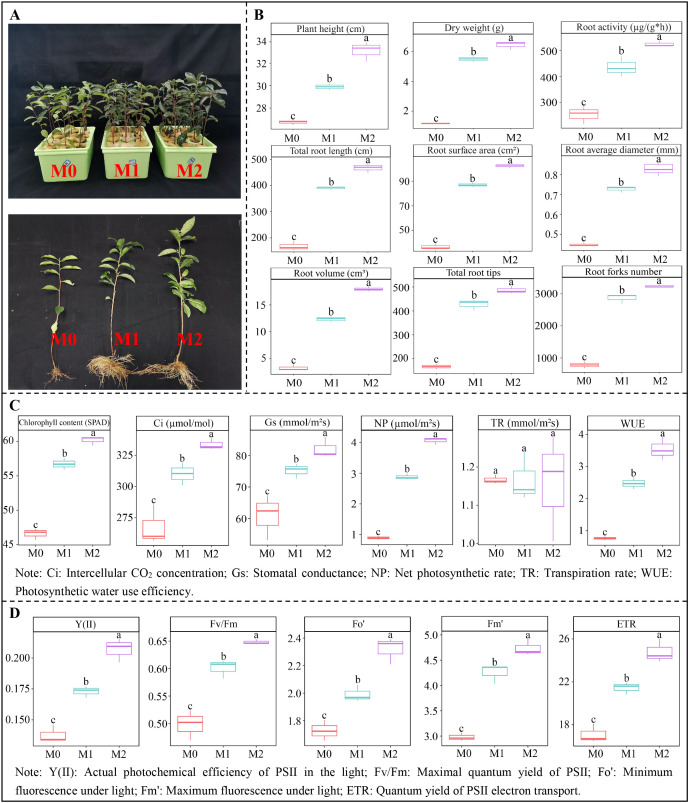
Effect of Mg regulation on growth and photosynthetic capacity of tea plant. M0, 0 mmol/L Mg ion. M1, 0.4 mmol/L Mg ion. M2, 0.8 mmol/L Mg ion. **(A)** Morphology of tea plant under Mg regulation. **(B)** Effect of Mg regulation on growth indexes of tea plant. **(C)** Effect of Mg regulation on photosynthetic physiological indexes of tea plant leaves. **(D)** Effect of Mg regulation on chlorophyll fluorescence parameters of tea plant leaves. Different lowercase letters indicate that the differences between samples reached the *p* < 0.05 level.

After different concentrations of Mg treatment, tea plant growth changed significantly as shown in **Figure 1A**. The analysis of tea plant growth indexes found (**Figure 1B**) that as Mg concentration increased (M0-M2), the plant height, dry weight, and root activity of tea plants treated with M2 were 1.24, 5.66, and 2.09 times higher than those treated with M0, respectively. The results of root scanning showed (**Figure 1B**) that with increasing Mg concentration (M0-M2), total root length, root average diameter, root surface area, root volume, root forks number, and total root tips of tea plants treated with M2 were 2.79, 1.89, 2.87, 5.82, 4.23, and 2.99 times higher than those treated with M0, respectively. The results suggested that Mg facilitated root growth and root vigor, thus promoted tea plant growth and biomass.

The analysis of photosynthetic physiological indexes revealed (**Figure 1C**) that as the Mg concentration increased (M0-M2), several key parameters exhibited a significant upward trend. Specifically, chlorophyll content, intercellular CO_2_ concentration (Ci), stomatal conductance (Gs), net photosynthetic rate (NP), photosynthetic water use efficiency (WUE) all increased significantly, and compared with M0 treatment, M2 treatment increased by 29.89%, 25.24%, 35.57%, 366.29%, and 373.68%, respectively. Notably the transpiration rate (TR) did not show a significant difference with increasing Mg concentration. The above findings reveal that Mg regulation has a positive impact on the photosynthetic physiological indexes of tea plants, potentially enhancing their photosynthetic efficiency and water use efficiency.

The analysis of fluorescence parameters indicated (**Figure 1D**) that when Mg concentration increased (M0-M2), several fluorescence parameters also increased significantly. Specifically, the actual photochemical efficiency of PSII in the light (Y(II)), maximal quantum yield of PSII (Fv/Fm), minimum fluorescence under light (Fo’), maximum fluorescence under light (Fm’), quantum yield of PSII electron transport (ETR) all showed an upward trend. And compared with M0 treatment, M2 treatment increased these parameters by 50.01%, 30.02%, 35.26%, 60.74%, and 46.02%, respectively. It is clear that Mg regulation has a beneficial effect on the photosynthetic physiological indexes and fluorescence parameters of tea plants. By improving these parameters, Mg regulation is likely to improve the photosynthesis capacity and facilitate their growth. This underscores during the cultivation of tea plants, adequate Mg nutrition is important to optimal growth and productivity.

### 3.2 Effects of Mg regulation on the antioxidant capacity and nutrient contents in roots and leaves of tea plant

**Figure 2:**
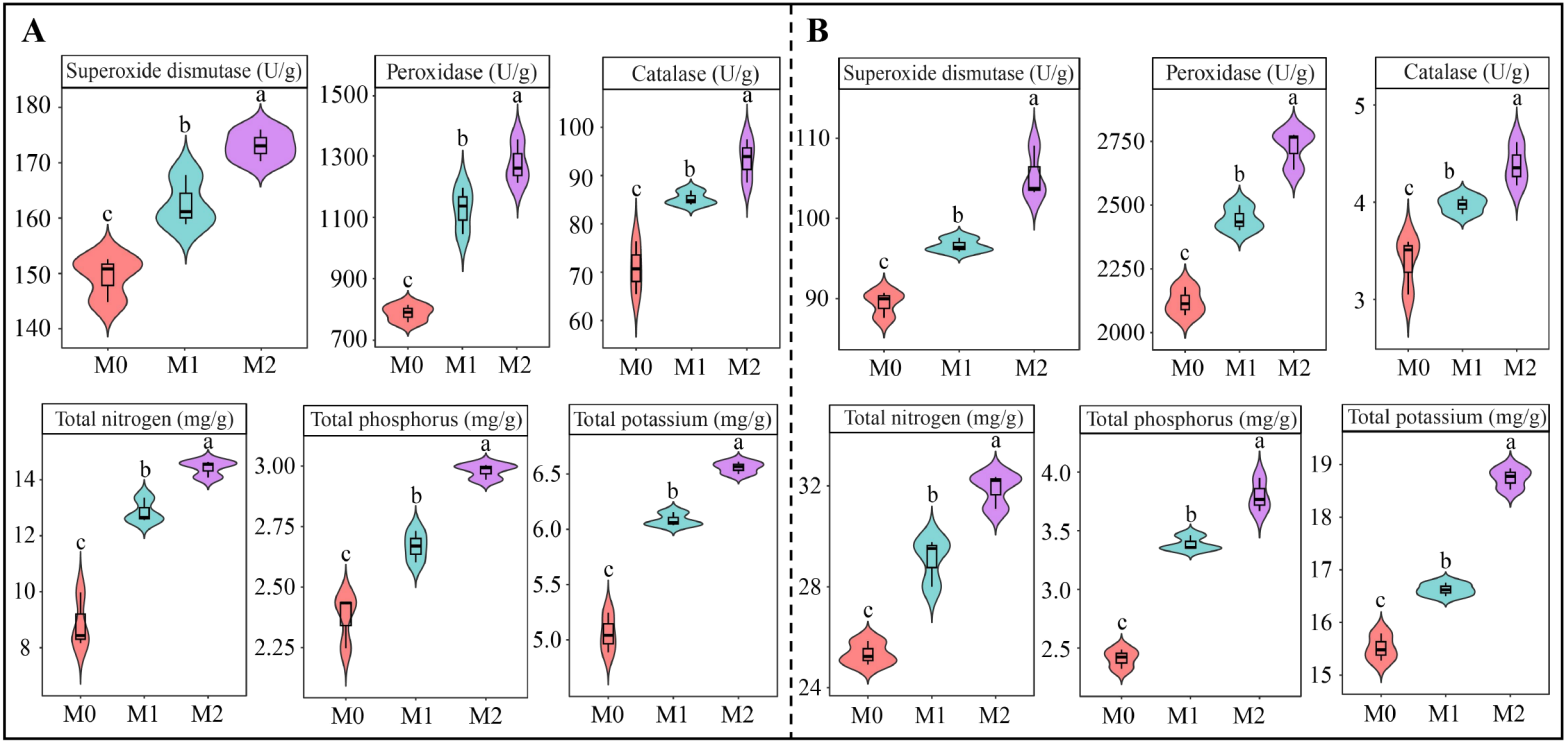
Effect of Mg regulation on the activity of antioxidant enzymes and nutrient content of tea plant roots and leaves. M0, 0 mmol/L Mg ion. M1, 0.4 mmol/L Mg ion. M2, 0.8 mmol/L Mg ion. **(A)** Effect of Mg regulation on the activity of antioxidant enzymes and nutrient content of tea leaves. **(B)** Effect of Mg regulation on the activity of antioxidant enzymes and nutrient content of tea plant roots. Different lowercase letters indicate differences between samples at *p* < 0.05.

After Mg regulation, there were significant changes in the antioxidant capacity and nutrient contents in both the roots and leaves of tea plants. Compared with M0 treatment, M2 treatment increased the activities of superoxide dismutase, peroxidase, and catalase in tea leaves by 32.99%, 64.17%, and 16.50%, respectively (**Figure 2A**). Additionally, the total nitrogen, potassium and phosphorus contents in the tea leaves exhibited a significant upward trend, increased by 64.45%, 30.63%, and 26.58%, respectively (**Figure 2A**). Furthermore, antioxidant capacity and nutrient contents in the tea plant’s root system also demonstrated a significant upward trend with increasing Mg concentration (**Figure 2B**). Specifically, compared with M0 treatment, M2 treatment increased the activities of peroxidase, superoxide dismutase, and catalase by 29.67%, 31.66%, and 18.80%, respectively. Similarly, the total nitrogen, phosphorus, and potassium contents increased by 26.91%, 59.34%, and 21.86%, respectively. These findings indicate that Mg is beneficial for enhancing protective enzyme activities in roots and leaves of tea plants, thereby improving their antioxidant capacity and nutrient absorption and accumulation abilities, ultimately promoting the growth of tea plants.

### 3.3 Effect of Mg regulation on quality indexes of tea leaves

**Figure 3:**
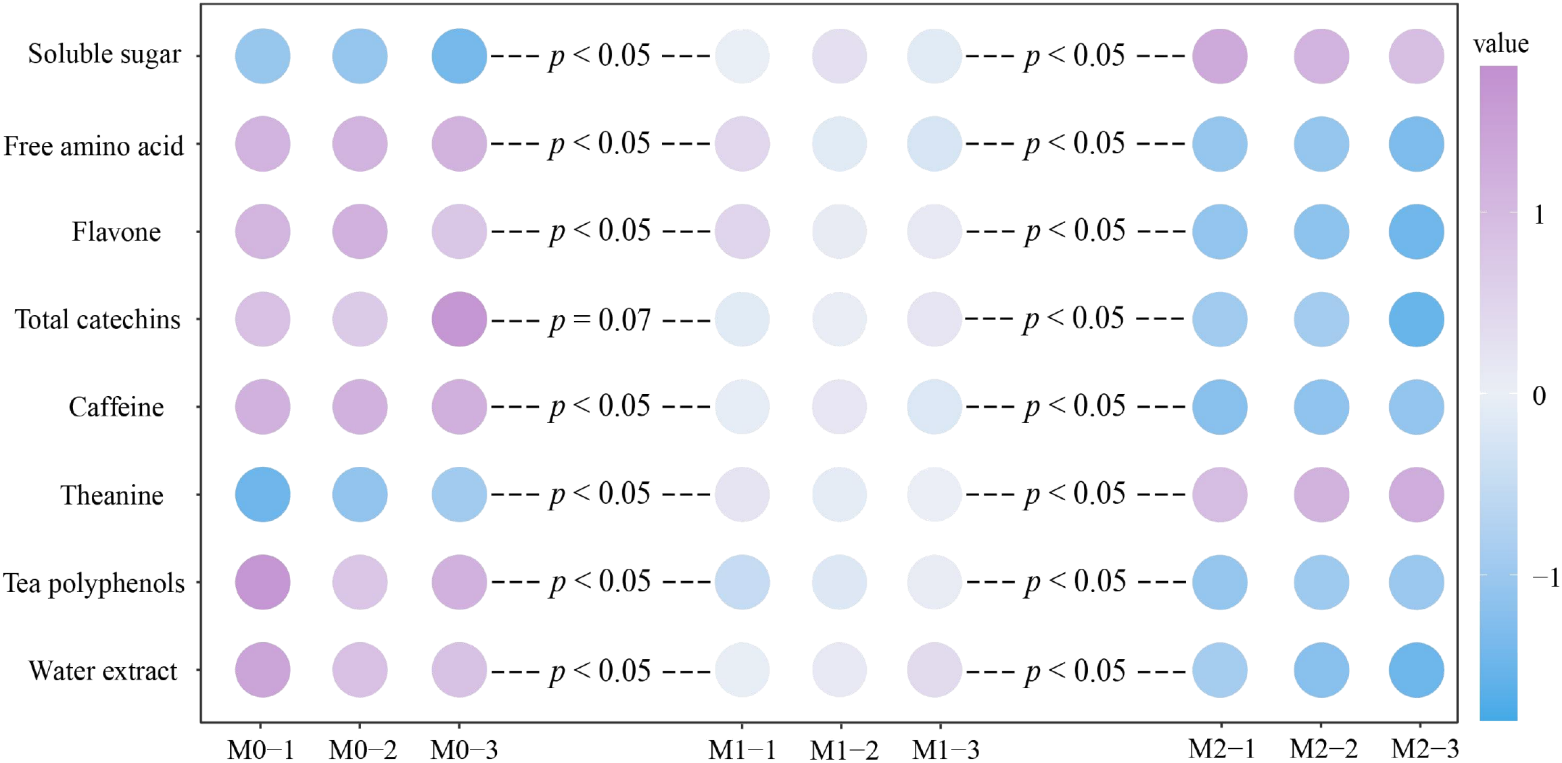
Effect of Mg regulation on tea quality. M0, 0 mmol/L Mg ion. M1, 0.4 mmol/L Mg ion. M2, 0.8 mmol/L Mg ion. Free amino acids are the content after deducting theanine.

The analysis of tea quality indexes revealed (**Figure 3**) that as Mg concentration increased (M0-M2), the contents of water extract, tea polyphenols, caffeine, total catechins, flavone and free amino acid (excluding theanine) in tea leaves exhibited a significant decrease. That is, compared with M0 treatment, M2 treatment reduced by 20.65%, 15.67%, 37.88%, 19.26%, 25.38%, and 39.84%, respectively. Conversely, the contents of theanine and soluble sugar presented a significant increase, and compared with M0 treatment, M2 treatment increased by 42.46% and 18.77%, respectively. It is evident that Mg significantly impacted the content of different quality indexes in tea leaves, which may consequently affect the overall quality of the tea.

### 3.4 Effect of Mg regulation on the content of hormones, hormone precursors and their derivatives in the roots and leaves of tea plants

**Figure 4:**
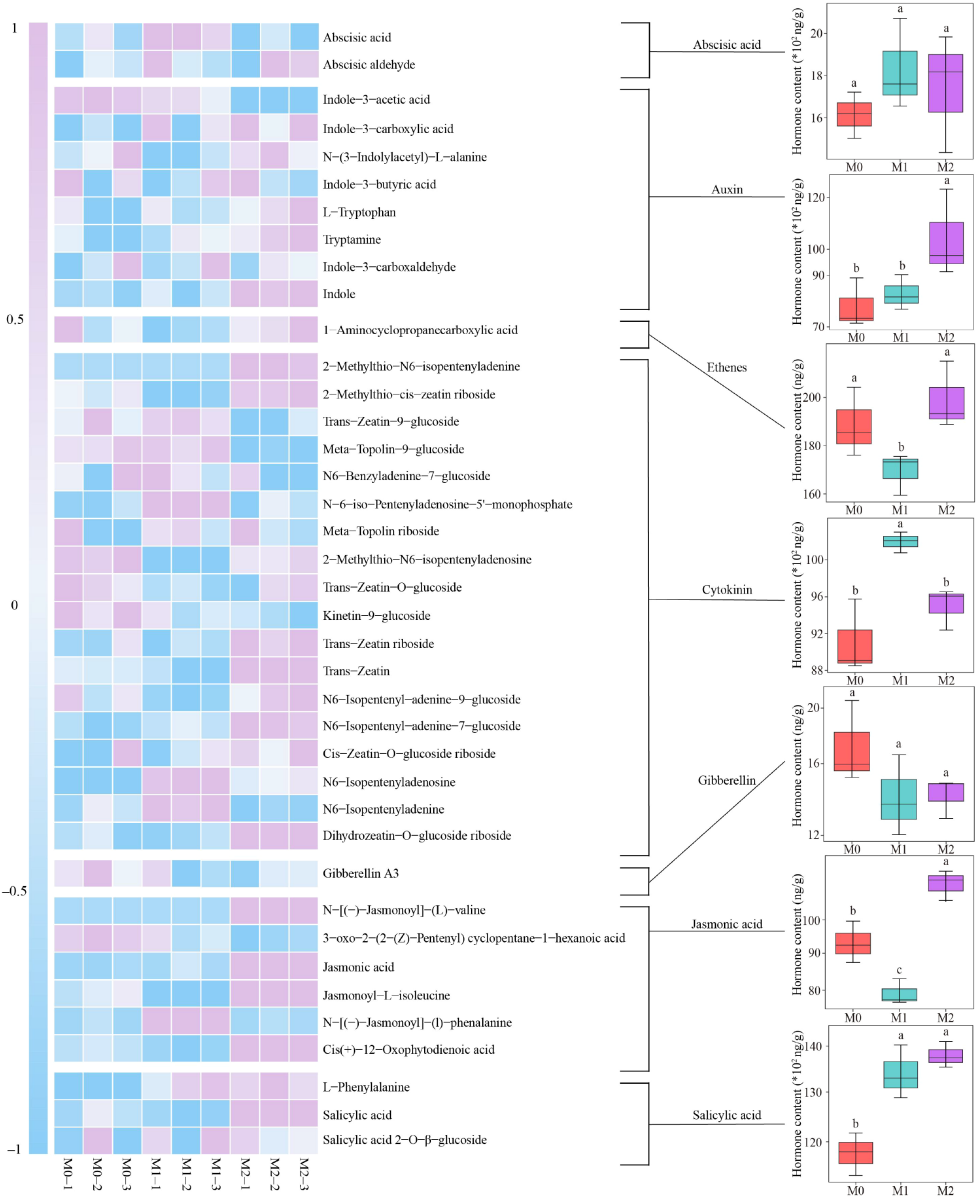
Effect of Mg regulation on the content of hormones, hormone precursors and their derivatives in tea plant leaves. M0, 0 mmol/L Mg ion. M1, 0.4 mmol/L Mg ion. M2, 0.8 mmol/L Mg ion. Different lowercase letters indicate differences between samples at the *p* < 0.05 level.

In this study, hormone metabolome technique was used to investigate the hormones, hormone precursors and their derivatives (General term: Hormones) present in the roots and leaves of tea plants, with a focus on analyzing how Mg impacted the content of different hormones. The findings from the hormone metabolome assay conducted on tea plant leaves were as follows (**Figure 4**). A total of 39 hormones were detected, which can be divided into seven groups, namely abscisic acid, auxin, ethenes, cytokinin, gibberellin, jasmonic acid and salicylic acid. Among them, M2 was significantly higher than M1 and M0 for auxin and jasmonic acid contents, M1 was significantly lower than M0 and M2 for ethenes, M1 was significantly higher than M0 and M2 for cytokinin, M1 and M2 were significantly higher than M0 for salicylic acid contents, and for abscisic acid and gibberellin contents, the differences between M0, M1 and M2 were not significant.

**Figure 5:**
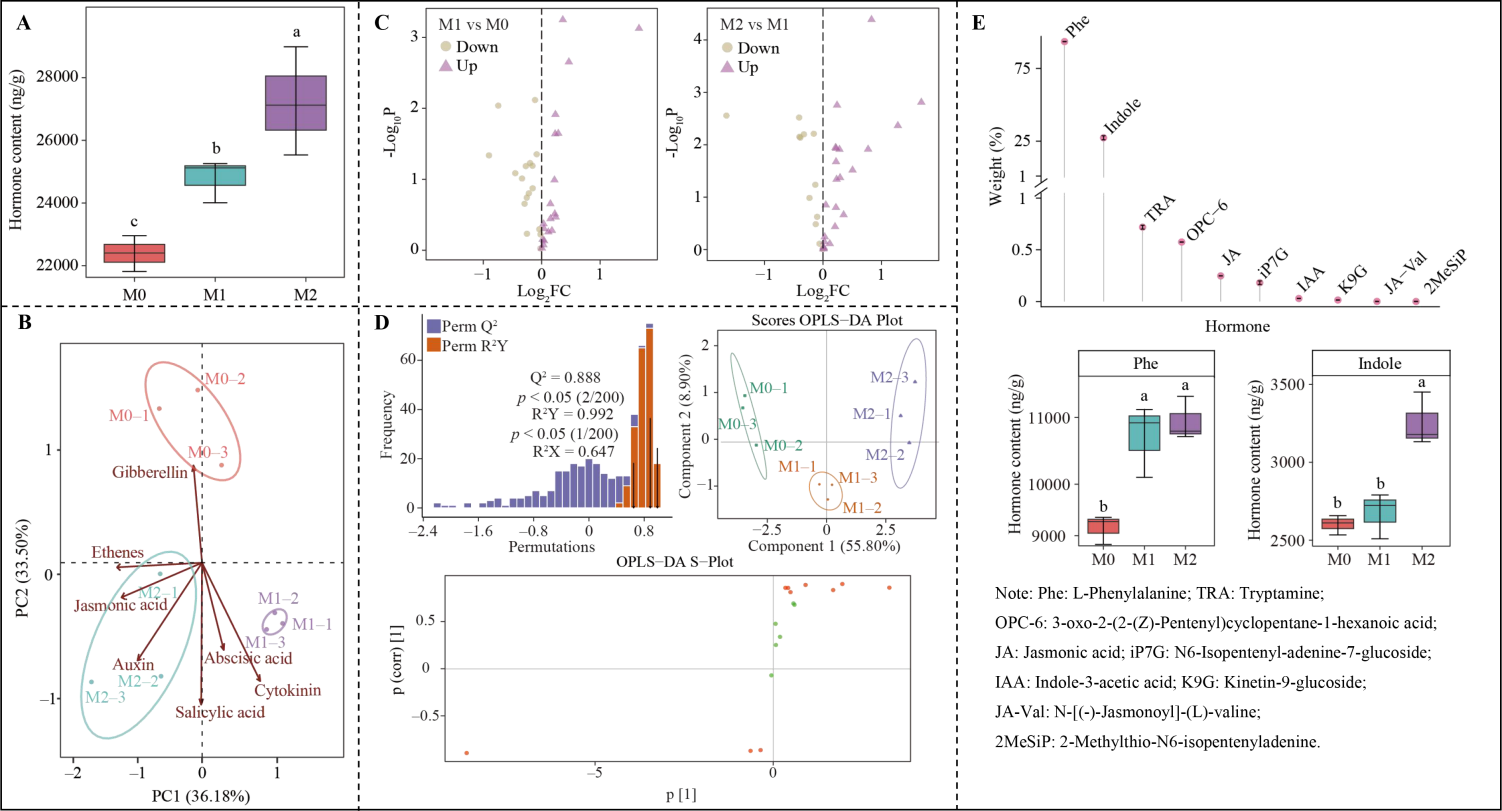
Screening for characteristic hormones that undergo significant changes in tea plant leaves under Mg regulation. M0, 0 mmol/L Mg ion. M1, 0.4 mmol/L Mg ion. M2, 0.8 mmol/L Mg ion. **(A)** Total hormone analysis of tea plant leaves. **(B)** PCA analysis of different hormone groups in tea plant leaves in response to Mg regulation. **(C)** Volcano plot screening for hormones with significant content changes in tea leaves response to Mg regulation. **(D)** Screening of key hormones with significant content changes in tea leaves response to Mg regulation using OPLS-DA modeling. **(E)** TOPSIS analysis of the weights of the key hormones and differential analysis of characteristic hormone contents in tea leaves response different Mg treatments. Different lowercase letters denote that the differences among different samples reached the significance level of *p* < 0.05.

In-depth analysis found (**Figure 5A**) that the total hormone content in tea plant leaves exhibited a significant increasing trend as Mg concentration increased (M0-M2), with an elevation from 22.39 μg/g to 27.21μg/g. PCA analysis demonstrated (**Figure 5B**) that the two principal components could effectively differentiate between M0, M1 and M2, contributing a total of 69.68% to the overall variance. It is evident that under different Mg concentrations, the hormone content in tea plant leaves underwent significant changes. Volcano plots were utilized for further analysis, revealing (**Figure 5C**) that 16 hormones showed significant alterations in their contents across different Mg concentrations, with 12 hormones increasing and 4 decreasing as Mg concentration rose. OPLS-DA was further employed to screen for significantly altered key hormones, resulting in (**Figure 5D**) the identification of 10 key hormones with importance eigenvalues (VIP) greater than 1. TOPSIS analysis was performed on these 10 key hormones to ascertain their contribution weights in distinguishing M0, M1, and M2, and to identify characteristic hormones. The results indicated (**Figure 5E**) that among the 10 key hormones, only 2 characteristic hormones, L-phenylalanine and indole, had contribution weights exceeding 1%. Specifically, for L-phenylalanine content, the difference from M1 and M2 was not significant, but both were significantly higher than M0. Conversely, for indole content, M2 was significantly greater than both M0 and M1, with no significant difference between M0 and M1. It is clear that the significant impact of Mg modulation on hormone levels in tea plant leaves, especially L-phenylalanine and indole.

**Figure 6:**
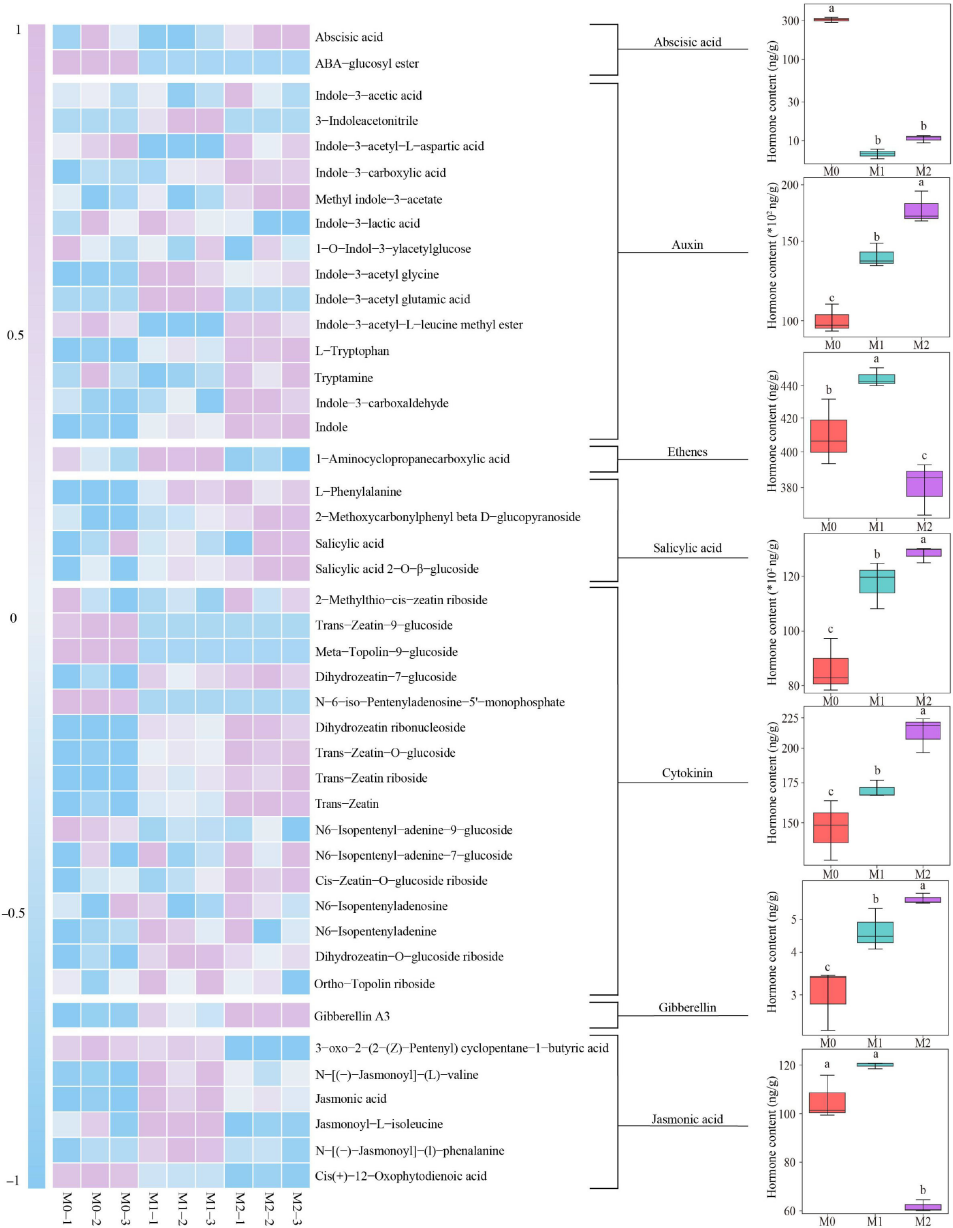
Effect of Mg regulation on the content of hormones, hormone precursors and their derivatives in tea plant roots. M0: Mg ion concentration 0 mmol/L. M1, Mg ion concentration 0.4 mmol/L. M2, Mg ion concentration 0.8 mmol/L. Different lowercase letters denote that the differences among different samples reached the significance level of *p* < 0.05.

The results of a hormone metabolome assay on the tea plant’s root system showed (**Figure 6**) that a total of 44 hormones were detected, which can be categorized into seven groups, namely, abscisic acid, auxin, ethenes, salicylic acid, cytokinin, gibberellin and jasmonic acid. Among these hormones, auxin, salicylic acid, cytokinin and gibberellin contents, showed a significant increase with increasing Mg concentration (M0-M2). For abscisic acid content, M0 was significantly greater than M1 and M2, while the difference between M1 and M2 was not significant. For ethenes content, M1 was significantly greater than M0 and M2, while M0 was significantly greater than M2. For jasmonic acid content, the difference between M0 and M1 was not significant, but both were significantly greater than M2.

**Figure 7:**
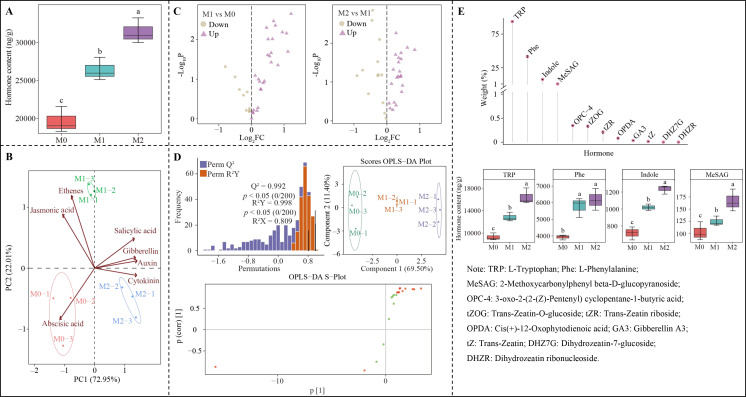
Screening for characteristic hormones that undergo significant changes in the tea plant’s root system under Mg regulation. M0, 0 mmol/L Mg ion. M1, 0.4 mmol/L Mg ion. M2, 0.8 mmol/L Mg ion. **(A)** Total hormone analysis of tea plant roots. **(B)** PCA analysis of different groups of hormones in tea plant roots response to Mg regulation. **(C)** Volcano plot screening for hormones with significant content changes in tea plant roots response to Mg regulation. **(D)** Screening of key hormones with significant content in tea plant roots response to Mg regulation using OPLS-DA modeling. **(E)** TOPSIS analysis of the weights of the key hormones and differential analysis of characteristic hormone contents in tea plant roots between different Mg treatments. Different lowercase letters denote that the differences among different samples reached the significance level of *p* < 0.05.

Further analysis found (**Figure 7A**) that as Mg concentration increased (M0-M2), the total amount of hormones in the tea plant’s root system showed a significant upward trend, i.e., from 19.64 μg/g to 31.38 μg/g. PCA analysis effectively distinguished between the different Mg concentrations (M0, M1, and M2) with an overall contribution of 94.96% (**Figure 7B**). Volcano plots were used to identify hormones with significant changes in their contents under different Mg concentrations (**Figure 7C**). A total of 20 hormones showed significant changes, with 16 hormones increasing and 4 hormones decreasing in their contents as Mg concentration increased. OPLS-DA was employed to screen key hormones significantly changed, and the results found (**Figure 7D**) that 12 key hormones with VIP greater than 1 were obtained. TOPSIS analysis of these 12 key hormones revealed (**Figure 7E**) that four characteristic hormones contributed more than 1% weight in differentiating M0, M1, and M2, namely, L-tryptophan, L-phenylalanine, indole and 2-methoxycarbonylphenyl beta-D-glucopyranoside. Among them, L-tryptophan, indole and 2-methoxycarbonylphenyl beta-D-glucopyranoside contents showed a significant increase with increasing Mg concentration. For L-phenylalanine content, the difference between M1 and M2 was not significant, but both were significantly higher than M0. It is clear that Mg regulation had a significant impact on the hormone content in the tea plant’s root system, with significant changes observed in four characteristic hormones.

### 3.5 Analysis of interaction effects

**Figure 8:**
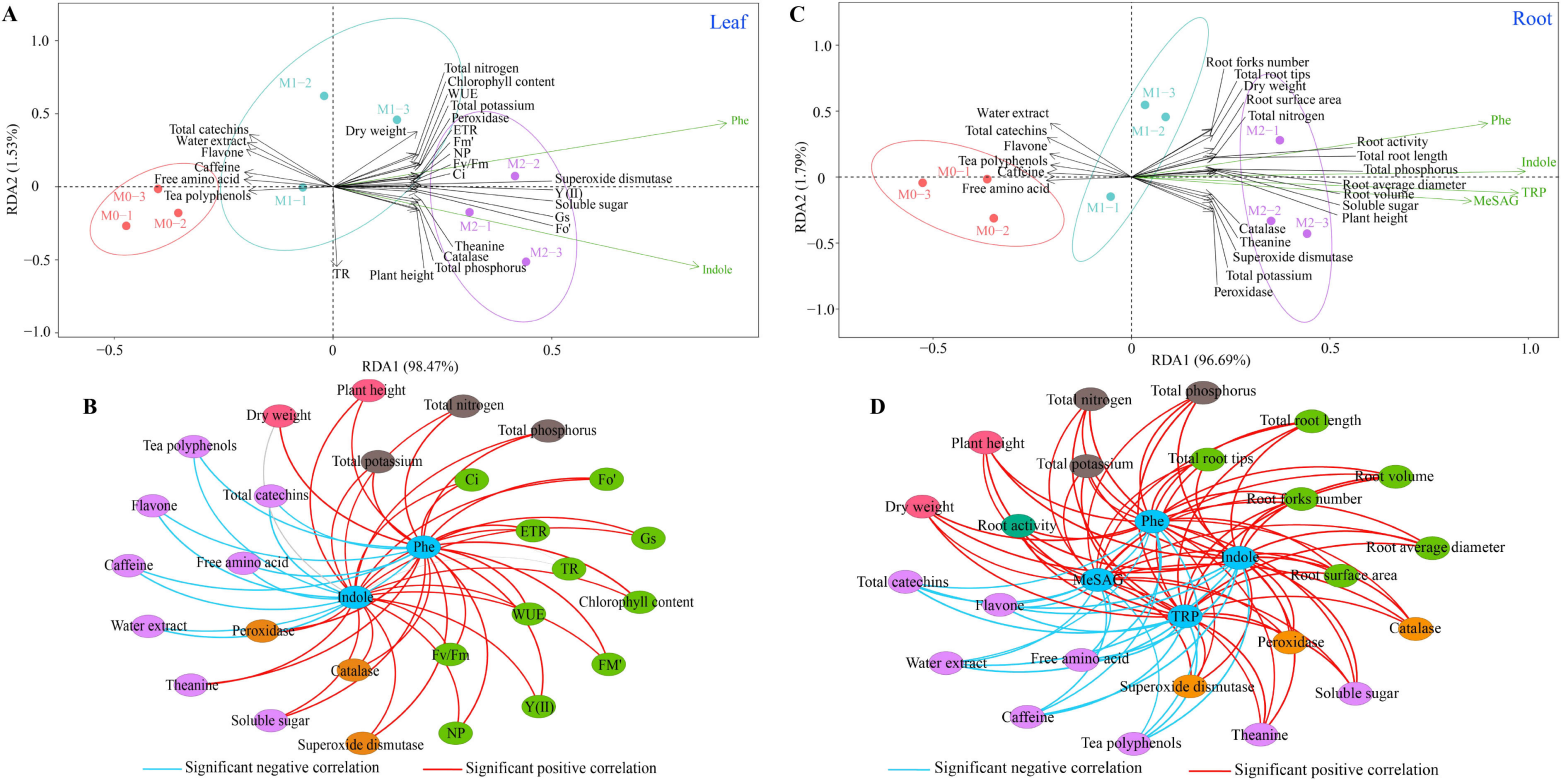
Interaction between characteristic hormones and growth indexes, quality indexes, photosynthesis indexes, antioxidant enzyme activities and nutrient contents of tea plant under Mg regulation. M0, 0 mmol/L Mg ion. M1, 0.4 mmol/L Mg ion. M2, 0.8 mmol/L Mg ion. Ci, Intercellular CO_2_ concentration. Gs, Stomatal conductance; NP, Net photosynthetic rate; TR, Transpiration rate; WUE, Photosynthetic water use efficiency; Y(II): Actual photochemical efficiency of PSII in the light. Fv/Fm, Maximal quantum yield of PSII. Fo’, Minimum fluorescence under light. Fm’, Maximum fluorescence under light. ETR, Quantum yield of PSIIelectron transport. **(A)** RDA plots of characteristic hormones of tea plant leaves and different indexes. **(B)** Interaction between characteristic hormones of tea plant leaves and different indexes. **(C)** RDA plots of characteristic hormones of tea plant roots and different indexes. **(D)** Interaction between the characteristic hormones of tea plant roots and different indexes.

Analysis of interaction effects between characteristic hormones and growth indexes, quality indexes, photosynthesis indexes, antioxidant capacity, and nutrient contents of tea plants under Mg regulation (**Figures 8A, B**) showed that these hormones exhibited significant positive correlation with plant height, dry weight, and photosynthesis physiological indexes, photosynthesis fluorescence parameters, antioxidant capacity, and nutrient contents. Additionally, these characteristic hormones of tea plant leaves showed significant positive correlations with certain tea leaf quality indexes, such as theanine and soluble sugar, while demonstrating significant negative correlations with others, including water extract, tea polyphenols, caffeine, total catechins, flavone and free amino acid. Furthermore, the examination of the interactions between characteristic hormones and different indexes in the tea plant’s root system under Mg regulation (**Figures 8C, D**) indicated that these hormones exhibited significant positive correlations with dry weight, root vigor, root growth indexes, antioxidant capacity, and nutrient contents. Similarly, characteristic hormones of the root system remained significant and positive correlations with the quality indexes of theanine and soluble sugar in tea leaves, while exhibiting significant negative correlations with water extract, tea polyphenols, caffeine, total catechins, flavone and free amino acid. These findings suggest that Mg regulation alters the synthesis of characteristic hormones in tea plants, ultimately influencing their growth and quality.

**Figure 9:**
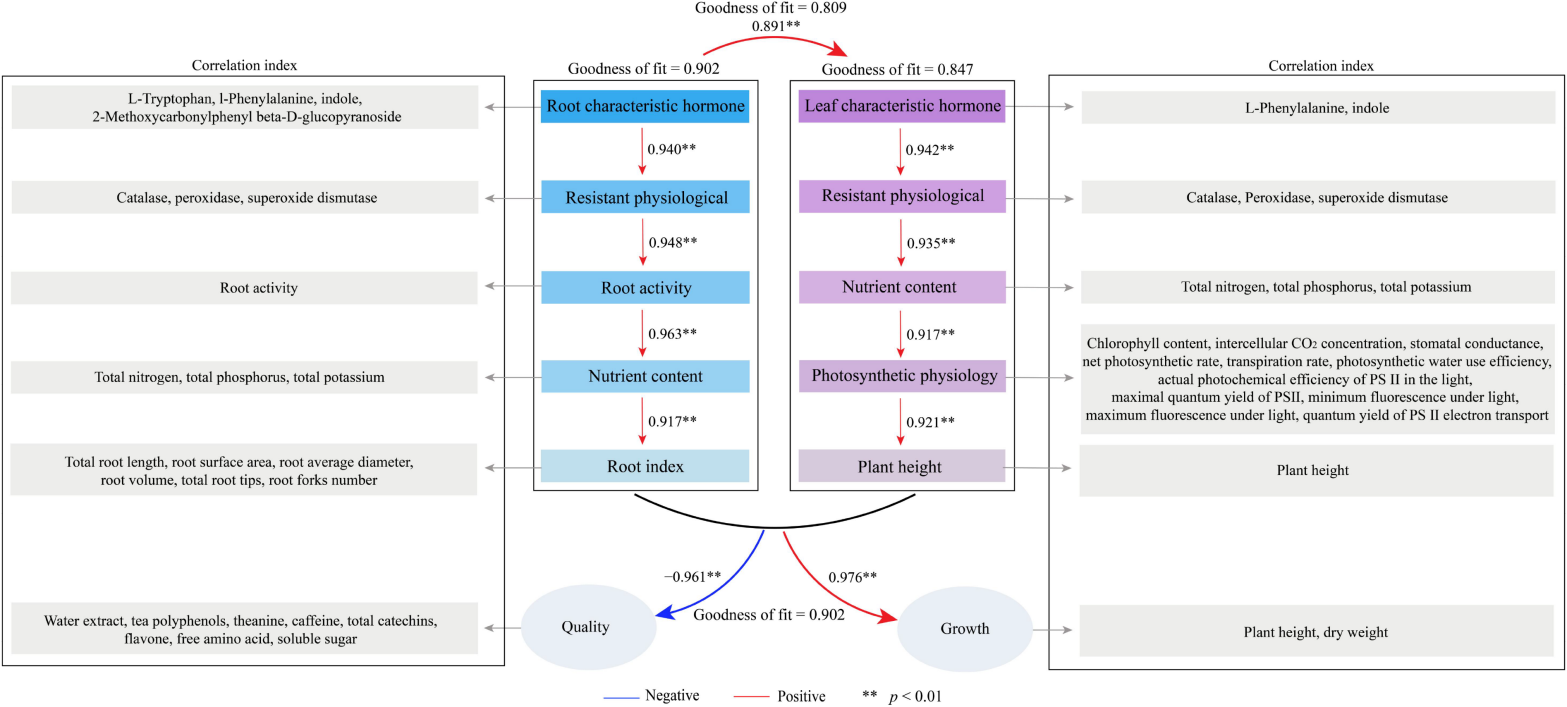
PLS-SEM equations analysis between characteristic hormones, growth indexes, quality indexes, photosynthesis indexes, antioxidant enzyme activities and nutrient contents of tea plant under Mg regulation.

The PLS-SEM equations were further constructed between different indexes under magnesium regulation to analyze the effects of characteristic hormones on different indexes and tea plant growth and quality. The results showed (**Figure 9**) that characteristic hormones in the root system of the tea plant positively regulated root antioxidant capacity (0.940**), root vigor (0.948**), nutrient accumulation capacity (0.963**), and root growth indexes (0.917*). However, characteristic hormones of tea plant leaves positively regulated leaf antioxidant capacity (0.942**), nutrient accumulation capacity (0.935**), photosynthesis capacity (0.917**) and tea plant height (0.921**). Overall changes in root system indexes positively regulated changes in different indexes of tea plant leaves (0.891**), while changes in overall indexes within the root system and leaves positively regulated tea plant growth (0.976**) and negatively regulated the formation of tea quality (-0.961**). It can be seen that magnesium regulation is favorable to promote the growth of tea plant, but unfavorable to the formation of tea quality.

### 3.6 Effect of exogenous treatments of characteristic hormones on the growth and quality of tea plants

**Figure 10:**
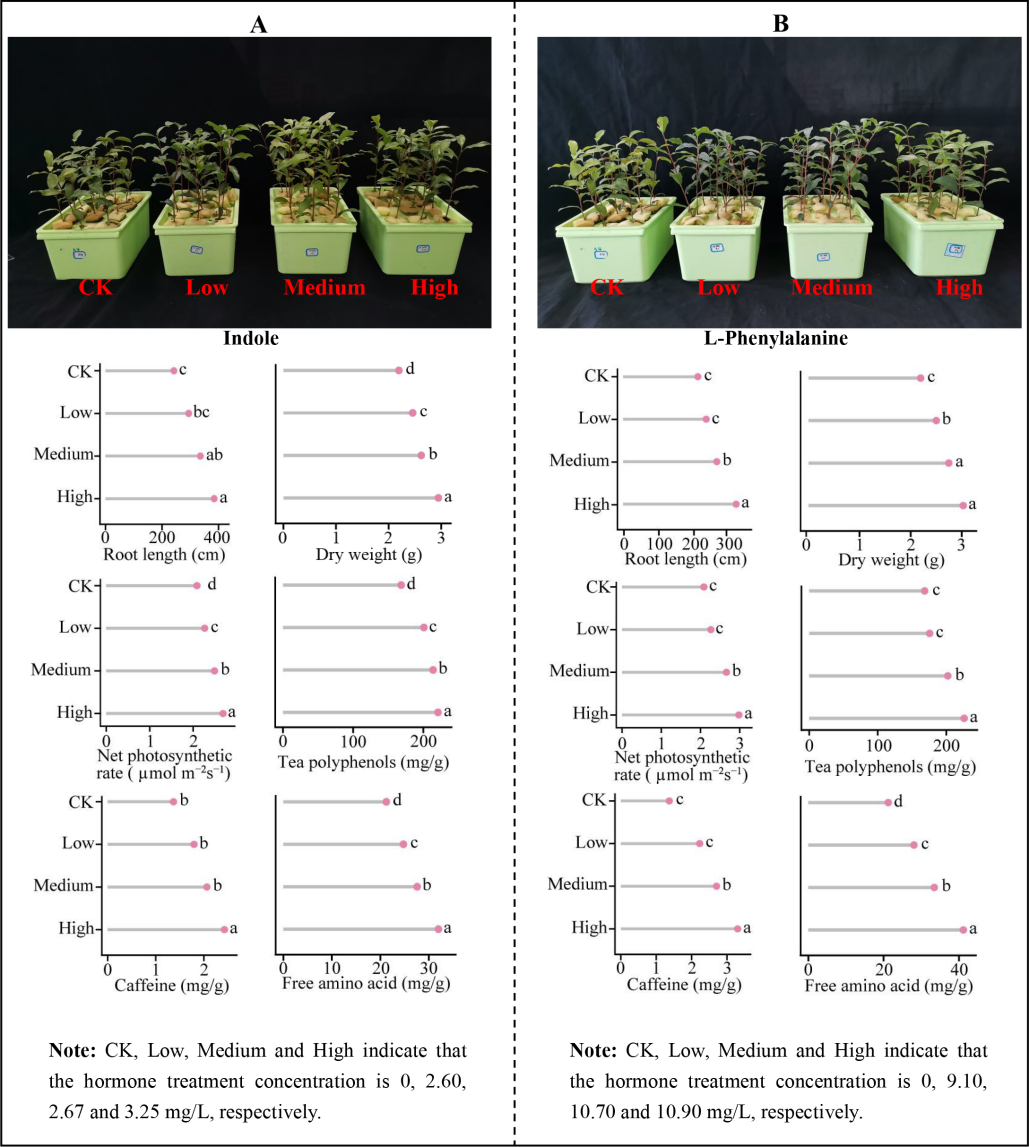
Effect of different concentrations of hormone sprays on growth indexes, net photosynthetic rate and quality of tea leaves. **(A)** Indole exogenous spraying treatment. **(B)** L-Phenylalanine exogenous spraying treatment. Different lowercase letters denote that the differences among different samples reached the significance level of *p* < 0.05; Free amino acids are the content after deducting theanine.

**Figure 11:**
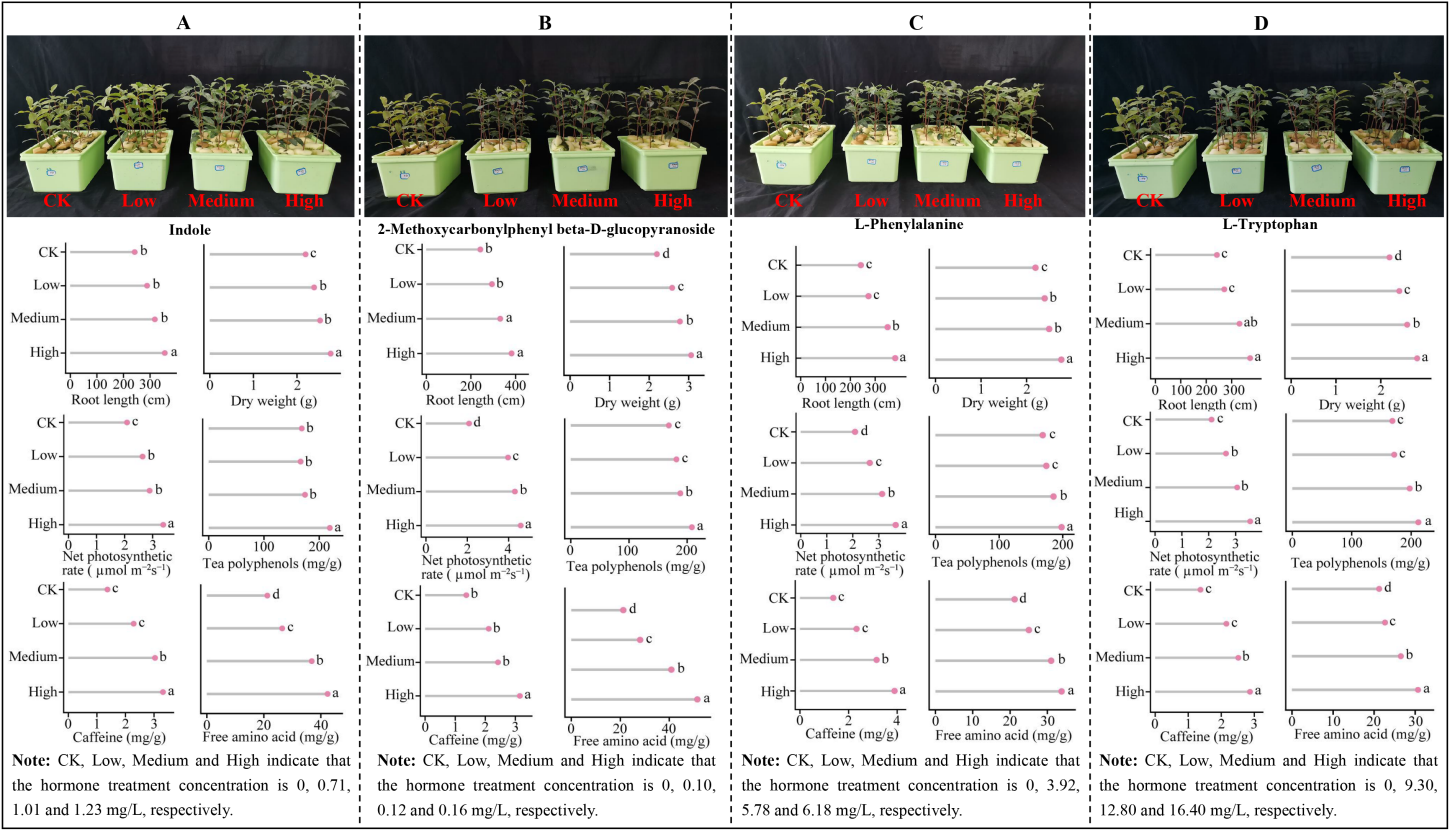
Effect of different concentrations of hormone additive treatments on growth indexes, net photosynthetic rate and quality of tea plant. **(A)** Indole exogenous treatment. **(B)** 2-Methoxycarbonylphenyl beta-D-glucopyranoside exogenous treatment. **(C)** L-Phenylalanine exogenous treatment. **(D)** L-Tryptophan exogenous treatment. Different lowercase letters indicate the differences between samples at *p* < 0.05 level; Free amino acids are the content after deducting theanine.

The above analysis revealed that the characteristic hormones significantly changed in tea leaves under Mg regulation were L-phenylalanine and indole, while the characteristic hormones significantly changed in the root system were L-tryptophan, L-phenylalanine, indole and 2-methoxycarbonylphenyl beta-D-glucopyranoside. As Mg concentration increased, these characteristic hormone contents rose, which facilitated tea plant growth but reduced the quality of tea leaves. In order to further explore the effects of these characteristic hormones on the growth and quality of tea plants, this study measured the concentrations of characteristic hormones detected in tea leaves and roots after treatment with different concentrations of Mg, and externally treated hydroponic tea plants to determine some key indexes related to tea plant growth and quality. When hydroponically grown tea plant leaves were treated with different concentrations of L-phenylalanine and indole through exogenous spray application, there was an upward trend observed in root length, dry weight, net photosynthetic rate, as well as tea polyphenol, caffeine, and free amino acid (excluding theanine) contents of tea leaves. This increasing trend was more pronounced as the concentration of hormone treatment increased (**Figures 10A, B**). Similarly, when different concentrations of L-tryptophan, L-phenylalanine, indole and 2-methoxycarbonylphenyl beta-D-glucopyranoside were exogenously added to the culture broth of hydroponically grown tea plants, there was also an increasing trend observed in root length, dry weight, net photosynthetic rate of tea plants and tea polyphenols, caffeine, and free amino acids (excluding theanine) contents of tea leaves. This increasing trend was more even obvious with the increase in hormone treatment concentration (**Figures 11A-D**). It can be seen that characteristic hormone exogenous treatment of tea leaves and root system could effectively promote tea plant growth, improve tea leaf quality, and with the increase of hormone concentration, the effect of promotion was more obvious.

## 4 Discussion

Mg plays a crucial role in various physiological and biochemical processes in plants, including chlorophyll synthesis, photosynthesis, respiratory metabolism, and nucleic acid metabolism, and is indispensable for plant growth (Ahmad et al., 2022; Hamedeh et al., 2022). Previous studies have shown that Mg promotes the differentiation and elongation of plant root cells, enhancing the surface area of the root system and improving the plant’s ability to absorb water and nutrients, which contributes to plant growth (Ghassemi-Golezani and Farhangi-Abriz, 2021). Mg also boosts the chlorophyll synthesis capacity of plants and enhances their photosynthetic capacity, further promoting plant growth (Farhat et al., 2016; Lv et al., 2024). Additionally, Mg increases the activity of protective enzymes in plants, thereby enhancing their environmental resistance and overall plant resistance (Ahmed et al., 2023). Mg further regulates the nutrient uptake capacity of plants, facilitating the accumulation and utilization of nutrients, ultimately promotes plant growth and increases yields (Potarzycki et al., 2022; Verma et al., 2021). The present study found that Mg significantly affects the physiological functions of tea plants, impacting their growth. As Mg concentration increased, the tea plant’s root system exhibited more vigorous growth, with significant increases in total root length, root surface area, and root volume. The activity of protective enzymes in the root system also increased, significantly enhancing root vigor. Consequently, the nutrient uptake and accumulation capacity of the tea plant’s root system improved. Furthermore, this study revealed that as Mg concentration increased, the activity of protective enzymes in tea plant leaves was enhanced, the accumulation capacity of nutrients rose, the photosynthetic physiological indexes and chlorophyll fluorescence parameters increased significantly, which effectively improved the photosynthetic capacity of tea plant and promoted tea plant growth, and plant height and dry weight increased significantly. However, it was also found in this study that with increasing Mg concentration, most of quality indexes, such as tea polyphenols, water extract, caffeine, total catechins, flavone and free amino acid showed a decreasing trend, except for theanine and soluble sugar. Livigni et al. (2019) found that while Mg promotes plant growth, excess Mg can hinder the synthesis of alkaloids, terpenoids and phenylpropanoid secondary metabolites. Liu et al. (2022c) found that at low Mg levels, the contents of water extract, tea polyphenols, caffeine, total catechins, and flavone in tea leaves were higher. Xu et al. (2021) reported that as Mg concentration increased, the sugar accumulation and transportation capacity in tea leaves increased. Similarly, Zhang et al. (2023a) observed that with increasing Mg concentration, the content of theanine and soluble sugar in tea leaves rose, while all other quality indexes decreased significantly. Mg plays a pivotal role in promoting the growth of the tea plant’s root system, enhancing its resistance, and improving the absorption and accumulation of nutrients by the roots. This, in turn, facilitates the accumulation of nutrients in the tea plant’s leaves, boosts its photosynthesis capacity, and fosters overall growth. However, as Mg concentration increases, the content of most quality indexes in the tea leaves to decrease, adversely affecting the quality of the tea leaves.

Endogenous hormones serve as crucial regulators of plant growth and development, present in low levels in plants yet exerting profound influence on plant growth and physiological metabolism, including physiological resistance, photosynthetic capacity, etc (Müller and Munné-Bosch, 2021). Mg plays a pivotal role in modulating plant hormone synthesis, and Mg deficiency often results in downregulated gene expression involved in plant hormone metabolic pathways, reduced hormone synthesis, and hindered plant growth (Du et al., 2024). Mao et al. (2022) revealed that Mg significantly boosts the content of endogenous plant hormones, particularly indole-3-acetic acid, zeatin, and gibberellin acid, while concurrently decreasing abscisic acid content. Zhang et al. (2023b) conducted a transcriptomic analysis of tea plant leaves to investigate Mg’s impact on gene expression and observed that as Mg concentration rose, there was a significant enhancement in gene expression related to the hormone metabolism pathway in tea plant leaves, leading to improved hormone synthesis. It was hypothesized that Mg would induce the alteration of hormone synthesis, ultimately influencing tea plant growth. Utilizing combined transcriptomic and metabolomic analysis, Li et al. (2024) discovered that Mg affects tea plant growth by regulating hormone synthesis, which in turn impacts substance synthesis and metabolic pathways within the tea plant. It is clear that Mg is intimately linked to tea plant hormone synthesis and growth. This study examined Mg’s influence on the hormone metabolome of tea plant roots and leaves, revealing a significant upward trend in total hormone content in both roots and leaves as Mg concentration increased. Mg regulation induced significant changes in two characteristic hormones, L-phenylalanine and indole, in tea plant leaves and four characteristic hormones, L-tryptophan, L-phenylalanine, indole, and 2-methoxycarbonylphenyl beta- D-glucopyranoside, in tea plant roots. L-phenylalanine, which belongs to the salicylic acid hormone family, serves as a precursor in the phenylpropane metabolic pathway. This pathway stimulates plant resistance, enhances plant environmental adaptation, and fosters plant growth (Dong and Lin, 2021; Zhu et al., 2023). Additionally, L-tryptophan is a vital precursor for the synthesizing several bioactive compounds in plants, such as indole and indole-3-acetic acid, which enhances the photosynthetic capacity of plants, increases their growth rate, and promotes plant growth (Tivendale and Millar, 2022; Bělonožníková et al., 2022). Sun et al. (2023) revealed that L-tryptophan content in the tea plant’s root system decreased, indole and indole-3-acetic acid content decreased significantly, and root growth of tea plant was weakened when Mg supply was insufficient. The effect of 2-methoxycarbonylphenyl beta-D-glucopyranoside on plant growth has not been reported. It is clear that as Mg concentration increased, characteristic hormone accumulated in the root system and leaves and promoted tea plant growth.

Furthermore, this study conducted an in-depth analysis of the interactions between characteristic hormones and different indexes using correlation network and PLS-SEM equations, and indicated that the root characteristic hormones of tea plants positively influenced root resistance, root vigor, nutrient accumulation capacity and root growth indexes, while the leaf characteristic hormones positively regulated leaf resistance, nutrient accumulation capacity, photosynthesis capacity and tea plant plant height. Overall changes in root system indexes positively regulated changes in different indexes of tea plant leaves, while changes in overall indexes within the root system and leaves positively regulated tea plant growth and negatively regulated the formation of tea quality. It is clear that Mg is favorable for promoting tea plant growth, but not for tea quality. The occurrence of this phenomenon may be significantly linked to alterations in the content of characteristic hormones within the root system and leaves. On this basis, this study further exogenously treated tea plants with characteristic hormones to investigate the effects of characteristic hormones on tea plant growth and quality. Exogenous spray treatment to hydroponically grown tea plant leaves using L-phenylalanine and indole, or exogenous addition treatment to tea plant culture fluid using L-tryptophan, L-phenylalanine, indole and 2-methoxycarbonylphenyl beta-D-glucopyranoside, all of which could effectively promote tea plant growth and quality. Clearly, increasing Mg concentration stimulated the synthesis of large quantities of hormones in both leaves and roots, which promoted plant growth. However, this came at the cost of reduced synthesis of several key tea quality components. And exogenous hormone treatment could effectively promote tea plant growth and quality. Therefore, the intensity of synthesis of characteristic hormones affected the tea leaf quality.

## 5 Conclusion

This study demonstrated that moderate application of Mg could effectively enhance the root vigor and protective enzyme activity of tea plants, promote root growth and development, enhance nutrient absorption capacity, thereby improving leaf photosynthesis efficiency, ultimately promoting tea plant growth and increasing biomass accumulation. However, the application of Mg showed inhibitory effects on the synthesis of some quality components in tea. Under the regulation of Mg, significant changes were observed in the content of various characteristic hormones in the roots (L-tryptophan, L-phenylalanine, indole, and 2-methoxycarbonylphenylbeta-D-glucopyranoside) and leaves (L-phenylalanine and indole) of tea plants. These hormones positively regulated the antioxidant enzyme activity, vigor, and growth status of roots, as well as the antioxidant enzyme activity, photosynthetic capacity, and plant growth of leaves. Overall, changes in root system indexes positively affected the physiological status of leaves, thereby promoting overall growth of tea plants, but had a negative impact on the formation of tea quality. Furthermore, exogenous application of these characteristic hormones was found to promote tea growth while improving tea quality, indicating that Mg might establish a trade-off between tea growth and quality formation by regulating hormone synthesis. Therefore, it is recommended to combine the appropriate application of Mg fertilizer with exogenous hormone treatment to achieve a synergistic improvement in tea yield and quality. This study provides new ideas and methods for balancing high yield and high quality in tea plantation cultivation and management.

## Data Availability

The original contributions presented in the study are included in the article/**Supplementary Material**. Further inquiries can be directed to the corresponding authors.
