# Supplementary material for: Response of characteristic hormones in tea roots and leaves under magnesium regulation and their balancing regulation on growth and quality
Source: Front Plant Sci. 2026 Jan 5;16:1703380. doi: 10.3389/fpls.2025.1703380 (PMC12812615; doi:10.3389/fpls.2025.1703380)
Supplement: Supplementary file 1 [file DataSheet1.pdf]

Supplementary Materials

Table S1 Standard curves and detection limits of different hormones

Index	Class	RT	Equation	r	LLOQ	ULOQ
ABA-glucosyl ester	ABA	4.44	y = 0.36598 x + 0.00740	0.993	5	500
Abscisic acid	ABA	5.27	y = 0.09856 x + 0.00133	0.995	0.1	500
Abscisic aldehyde	ABA	5.49	y = 0.03505 x + 0.00414	0.999	5	500
L-tryptophan	Auxin	3.36	y = 0.00920 x + 0.00351	0.999	2	10000
Tryptamine	Auxin	3.62	y = 0.14425 x - 0.00375	0.998	0.1	500
2-Oxindole-3-acetic acid	Auxin	4.22	y = 0.01197 x + 0.00113	0.999	1	500
1-O-Indol-3-ylacetylglucose	Auxin	4.27	y = 0.00354 x + 7.45427e^{-4}	0.992	5	10000
Indole-3-acetyl-L-aspartic acid	Auxin	4.28	y = 0.01611 x + 3.09370e^{-5}	1.000	0.1	500
3-Indole acetamide	Auxin	4.32	y = 0.73510 x + 0.01301	0.991	0.1	500
Indole-3-acetyl glutamic acid	Auxin	4.39	y = 0.19084 x + 0.00126	0.999	0.1	500
Indole-3-acetyl glycine	Auxin	4.41	y = 0.38674 x + 0.00671	0.999	1	500
Indole-3-lactic acid	Auxin	4.63	y = 0.01873 x + 0.03305	1.000	2	500
N-(3-Indolylacetyl)-L-alanine	Auxin	4.71	y = 0.29866 x + 0.00169	1.000	0.1	500
Indole-3-carboxylic acid	Auxin	4.76	y = 0.00915 x + 4.26196e^{-4}	0.999	0.5	500
Indole-3-carboxaldehyde	Auxin	4.91	y = 0.05873 x + 0.00345	0.992	0.1	500
Indole-3-acetic acid	Auxin	5.03	y = 0.01616 x + 6.03873e^{-4}	0.999	0.2	500
3-Indoleacrylic acid	Auxin	5.24	y = 0.01589 x + 3.67271e^{-4}	0.998	0.2	500
N-(3-Indolylacetyl)-L-valine	Auxin	5.38	y = 1.53831 x - 0.00473	0.999	0.1	500
3-Indolepropionic acid	Auxin	5.45	y = 0.04592 x + 9.29204e^{-4}	0.999	0.1	500
Indole-3-acetyl-L-glutamic acid dimethyl ester	Auxin	5.55	y = 0.06970 x - 0.00415	1.000	0.5	500
Indole-3-acetyl-L-tryptophan	Auxin	5.71	y = 0.46427 x + 3.97415e^{-4}	1.000	0.1	500
N-(3-Indolylacetyl)-L-leucine	Auxin	5.75	y = 1.88393 x + 0.01697	1.000	0.1	500
Indole-3-butyric acid	Auxin	5.76	y = 0.03265 x + 0.02120	0.999	0.2	500

3-Indoleacetonitrile	Auxin	5.8	$y = 0.03343x + 0.00262$	0.999	0.5	500
N-(3-Indolylacetyl)-L-phenylalanine	Auxin	5.83	$y = 0.75483x + 0.00383$	0.999	0.5	500
Methyl indole-3-acetate	Auxin	6.09	$y = 0.07387x + 6.82104e^{-5}$	0.996	0.2	500
Indole-3-acetyl-L-valine methyl ester	Auxin	6.1	$y = 3.10195x + 0.07398$	0.999	0.1	500
Indole-3-acetyl-L-leucine methyl ester	Auxin	6.44	$y = 3.86036x + 0.00832$	0.994	0.1	500
Indole-3-acetyl-L-phenylalanne methyle ester	Auxin	6.48	$y = 2.98662x + 0.00441$	0.992	0.1	500
Indole	Auxin	N/A	$y = 0.00546x + 0.00300$	0.999	0.5	500
9-Ribosyl-trans-zeatin 5'-monophosphate	CK	2.59	$y = 0.01311x - 0.00606$	0.995	2	500
trans-Zeatin-O-glucoside	CK	3.01	$y = 0.11058x + 0.00142$	0.999	0.2	500
cis-Zeatin riboside monophosphate	CK	3.06	$y = 0.01512x - 8.08952e^{-4}$	0.997	2	500
trans-Zeatin	CK	3.1	$y = 0.15080x + 0.01283$	0.996	0.1	500
trans-Zeatin-9-glucoside	CK	3.18	$y = 0.06770x + 0.00336$	0.997	1	500
Dihydrozeatin	CK	3.19	$y = 0.06564x + 0.00229$	0.999	0.1	500
Dihydrozeatin-7-glucoside	CK	3.19	$y = 0.48700x + 0.00133$	0.991	0.1	500
cis-Zeatin	CK	3.25	$y = 0.04919x - 6.85505e^{-4}$	0.999	0.1	500
cis-Zeatin-9-glucoside	CK	3.3	$y = 0.47153x - 2.71748e^{-4}$	0.994	0.1	500
Dihydrozeatin-O-glucoside riboside	CK	3.48	$y = 0.44287x + 0.00134$	1.000	0.1	500
cis-Zeatin-O-glucoside riboside	CK	3.5	$y = 0.05950x - 0.02576$	0.994	0.1	500
para-Topolin	CK	3.55	$y = 0.06113x + 5.89174e^{-5}$	0.998	0.2	500
4-[[(9-beta-D-Glucopyranosyl-9H-purin-6-yl)amino]methyl]phenol	CK	3.56	$y = 0.09044x - 2.47945e^{-4}$	0.991	0.1	500
trans-Zeatin riboside	CK	3.59	$y = 0.16226x + 4.22331e^{-4}$	0.994	0.1	500
Dihydrozeatin ribonucleoside	CK	3.6	$y = 0.06702x + 3.04370e^{-4}$	0.992	0.1	500
cis-Zeatin riboside	CK	3.67	$y = 0.16028x + 7.44328e^{-4}$	0.990	0.1	500
N6-Isopentenyl-adenine-7-glucoside	CK	3.69	$y = 0.28608x + 5.89397e^{-4}$	0.992	0.1	500
meta-Topolin	CK	3.72	$y = 0.19318x + 8.00060e^{-4}$	0.992	2	500
meta-Topolin-9-glucoside	CK	3.73	$y = 0.09686x + 4.40456e^{-4}$	0.990	0.1	500

N6-Benzyladenine-7-glucoside	CK	3.79	$y = 0.37163 x + 6.51142e^{-4}$	0.990	0.1	500
N-6-iso-Pentenyladenosine-5'-monophosphate	CK	3.79	$y = 0.03993 x - 0.00182$	0.996	1	500
Kinetin	CK	3.81	$y = 0.08135 x + 3.13369e^{-4}$	0.993	0.1	500
Kinetin-9-glucoside	CK	3.81	$y = 0.20963 x + 0.00129$	0.991	0.1	500
para-Topolin riboside	CK	3.93	$y = 0.18283 x + 7.17201e^{-4}$	0.993	0.1	500
N6-Isopentenyl-adenine-9-glucoside	CK	4.08	$y = 0.20839 x + 1.88324e^{-4}$	0.991	0.1	500
ortho-Topolin-9-glucoside	CK	4.09	$y = 0.07441 x + 1.05846e^{-4}$	0.990	0.1	500
meta-Topolin riboside	CK	4.09	$y = 0.19867 x + 1.50585e^{-4}$	0.991	0.1	500
N6-Isopentenyladenine	CK	4.11	$y = 0.04972 x + 0.00304$	1.000	0.1	500
ortho-Topolin	CK	4.14	$y = 0.24939 x + 0.00152$	0.990	0.1	500
Kinetin riboside	CK	4.22	$y = 0.29736 x + 6.26829e^{-4}$	0.990	0.1	500
N6-Benzyladenine-9-glucoside	CK	4.24	$y = 0.28481 x + 0.00114$	0.990	0.1	500
6-Benzyladenine	CK	4.29	$y = 0.26469 x + 0.00207$	0.990	0.1	500
2-Chloro-trans-zeatin	CK	4.41	$y = 0.13387 x + 4.95519e^{-4}$	1.000	0.1	500
ortho-Topolin riboside	CK	4.46	$y = 0.15636 x + 7.28751e^{-4}$	0.990	0.1	500
N6-Isopentenyladenosine	CK	4.49	$y = 0.12768 x + 9.61159e^{-4}$	0.990	0.1	500
2-Methylthio-cis-zeatin	CK	4.5	$y = 0.03266 x + 4.39415e^{-5}$	0.993	0.1	500
2-Methylthio-cis-zeatin riboside	CK	4.56	$y = 0.12288 x + 0.00158$	0.998	0.1	500
6-Benzyladenosine	CK	4.64	$y = 0.48590 x + 7.05284e^{-4}$	0.990	0.1	500
2-Methylthio-N6-isopentenyladenosine	CK	5.75	$y = 0.04131 x - 2.83464e^{-5}$	0.991	0.1	500
2-Methylthio-N6-isopentenyladenine	CK	5.86	$y = 0.02959 x + 2.23593e^{-5}$	0.996	0.1	500
1-Aminocyclopropanecarboxylic acid	ETH	0.72	$y = 14688.31351 x + 11116.13700$	0.993	1	500
Gibberellin A8	GA	3.81	$y = 0.10438 x + 9.84937e^{-4}$	0.995	2	500
Gibberellin A29	GA	3.97	$y = 0.00507 x + 0.00114$	0.999	2	500
Gibberellin A3	GA	4.42	$y = 0.06229 x + 0.00270$	0.999	1	500
Gibberellin A1	GA	4.46	$y = 0.02298 x + 0.00299$	0.997	2	500

Gibberellin A6	GA	4.87	y = 0.07968 x + 0.00581	0.998	1	500
Gibberellin A19	GA	5.13	y = 0.02358 x + 0.00497	0.997	5	500
Gibberellin A5	GA	5.26	y = 0.03046 x + 0.00700	0.998	5	500
Gibberellin A20	GA	5.33	y = 0.01347 x + 0.00401	0.995	2	500
Gibberellin A44	GA	5.37	y = 0.03298 x - 0.00385	0.994	5	500
Gibberellin A34	GA	5.53	y = 0.09218 x - 0.00108	0.990	2	500
Gibberellin A51	GA	5.66	y = 0.01503 x + 0.00134	0.993	5	500
Gibberellin A53	GA	5.71	y = 0.04979 x + 0.00305	0.994	2	500
Gibberellin A7	GA	6.07	y = 0.30152 x + 0.01089	0.997	1	500
Gibberellin A4	GA	6.15	y = 0.05009 x + 0.00752	0.996	2	500
Gibberellin A24	GA	6.33	y = 0.08050 x + 8.30600e^{-4}	0.994	2	500
Gibberellin A15	GA	6.87	y = 0.03439 x + 7.53522e^{-4}	0.993	2	500
Gibberellin A9	GA	6.88	y = 0.08212 x + 0.00447	0.993	2	500
Gibberellin A12 aldehyde	GA	7.73	y = 0.04992 x + 6.79460e^{-4}	0.996	5	500
12-Hydroxyjasmonic acid	JA	4.76	y = 0.05926 x + 0.01781	0.998	5	500
Jasmonate-1-aminocyclopropane-1-carboxylic acid	JA	5.32	y = 0.09447 x - 4.75448e^{-4}	0.999	0.5	500
Jasmonic acid	JA	5.79	y = 0.02691 x + 9.20212e^{-4}	0.999	0.2	500
N-[(-)-Jasmonoyl]-(L)-valine	JA	5.98	y = 0.35645 x + 1.36136e^{-5}	0.997	0.1	500
Dihydrojasmonic acid	JA	6.15	y = 0.02957 x + 0.00231	0.995	1	10000
Jasmonoyl-L-isoleucine	JA	6.32	y = 0.09771 x + 4.19350e^{-4}	0.999	0.1	500
N-[(-)-Jasmonoyl]-(l)-phenalanine	JA	6.37	y = 0.32243 x + 5.18045e^{-4}	0.996	0.1	500
3-oxo-2-(2-(Z)-Pentenyl) cyclopentane-1-butyric acid	JA	6.39	y = 0.01991 x + 0.01384	0.997	2	500
Methyl jasmonate	JA	6.93	y = 0.67770 x + 0.03505	1.000	0.2	500
3-oxo-2-(2-(Z)-Pentenyl)cyclopentane-1-hexanoic acid	JA	7.02	y = 0.05035 x + 1.72126e^{-4}	0.999	5	500
cis(+)-12-Oxophytodienoic acid	JA	7.26	y = 0.93183 x + 0.00974	0.994	0.1	500
Melatonine	MLT	4.87	y = 1.60078e6 x + 7642.95763	0.995	0.5	500

L-Phenylalanine	SA	2.07	y = 844.12729 x + 12428.79365	0.990	1	15000
Salicylic acid 2-O-β-glucoside	SA	3.57	y = 0.04494 x + 8.46878e^{-4}	0.992	1	10000
2-Methoxycarbonylphenyl beta-D-glucopyranoside	SA	4.13	y = 3.28990e4 x + 6.59920e^{4}	0.991	20	500
2-Coumarate	SA	4.87	y = 5.14529e4 x + 3.09437e^{4}	0.994	10	500
Salicylic acid	SA	5.08	y = 0.11766 x + 0.01018	1.000	0.5	500
trans-Cinnamic acid	SA	5.56	y = 149.83510 x + 179.18753	0.994	200	10000
（±）Strigol	SL	6.38	y = 1040.29721 x - 1192.15665	0.999	20	10000
5-Deoxystrigol	SL	7.51	y = 19646.82487 x - 2040.97858	0.996	2	10000

Note: Index: Hormone name; Class: Classification of hormones; RT: Retention time; Equation: Linear equation; r: Coefficient of Correlation; LLOQ: Lower limit of quantitation (ng/mL); ULOQ: Higher limit of quantitation (ng/mL).
